# Examining the episodic context account: does retrieval practice enhance memory for context?

**DOI:** 10.1186/s41235-019-0202-3

**Published:** 2019-12-18

**Authors:** Min Kyung Hong, Sean M. Polyn, Lisa K. Fazio

**Affiliations:** 1 Department of Psychology and Human Development, Vanderbilt University, 230 Appleton Pl #552, Nashville, TN 37203 USA; 2 Department of Psychology, Vanderbilt University, Nashville, TN USA

**Keywords:** Retrieval practice, Testing effect, Context, Memory

## Abstract

Retrieval practice, such as filling in blanks or taking quizzes, is firmly established as an effective study strategy. However, the underlying mechanism of how retrieval practice benefits memory is still unclear. One current theory, the episodic context account, proposes that retrieval enhances memory by reinstating a prior learning context. This retrieved context is then strengthened and updated to include context at the time of recall, which later serves as an effective retrieval cue. However, few studies have directly tested this hypothesis. We did so by examining participants’ memory for the initial study context. Across three experiments, participants encoded cue-target pairs presented in different colors and either restudied or practiced retrieving the targets. If retrieval practice benefits memory by reinstating the prior episodic context, participants who successfully retrieved the items during practice should have enhanced memory for context details (i.e. font color) compared to participants who restudied the pairs. Contrary to this prediction, memory for font colors did not differ between the restudy condition and the retrieval practice condition. Even when font color was actively attended to and integrated with the to-be-remembered items, retrieval practice did not increase memory for this aspect of context. Our results suggest that the context reinstated during retrieval practice is limited in nature. Aspects of the context that are not essential to retrieval of the item are not strengthened by retrieval practice.

## Significance statement

Retrieval practice has been shown to be an effective learning strategy as it improves long-term memory retention more than other strategies such as restudying. While it is assumed that the act of retrieval changes memory in a way that leads to this benefit, the exact mechanism remains unclear. Yet, a better understanding of the mechanism behind the benefits of retrieval practice will allow students and teachers to optimize their use of the technique and further improve student learning. Across three experiments, we explore one possible mechanism - that retrieval practice improves memory by reinstating and strengthening the original study context. To test this prediction, we examined if retrieval practice improves memory for contextual details from the study phase more than restudying. We found no evidence that retrieval practice improves memory for details of the original study context. If the benefits of retrieval practice are due to reinstating and strengthening the original study context, then that reinstated context does not include details such as font color.

## Examining the episodic context account: does retrieval practice enhance memory for prior context?

The benefits of retrieval practice on subsequent memory are firmly established (see Rowland, 2014 for a review). This benefit, often referred to as retrieval-based learning or the testing effect, has been found consistently in both laboratory and classroom contexts (e.g. McDaniel, Agarwal, Huelser, McDermott, & Roediger III, 2011; Roediger & Karpicke, 2006). However, the underlying mechanism is still unclear. With every act of retrieval, it is assumed that there is *some* change that improves one’s ability to retrieve that knowledge in the future. The exact nature of that change, however, is still unknown. Several theories have been proposed to explain the effect of retrieval practice on learning (e.g., Bjork, 1994; Carpenter & Delosh, 2006; Hopper & Huber, 2018; Pyc & Rawson, 2009; Rickard & Pan, 2017), but none are currently widely accepted by the field.

The episodic context account, proposed by Karpicke, Lehman, and Aue (2014), is one recent theory of why retrieval practice is beneficial. The authors suggest that the mnemonic benefit of retrieval stems from reinstating the episodic context associated with the target (or to-be-retrieved) memory. The account builds upon current context-based memory models of encoding and retrieval (e.g. Howard & Kahana, 2002; Lehman & Malmberg, 2013; Mensink & Raaijmakers, 1988). These models suggest that when people encode an event, they encode both the event itself and the context associated with the event. Later retrieval attempts are then facilitated by retrieving the context of the study episode and using it to guide memory search.

Karpicke et al. (2014) expand these context-based models to explain the benefits of retrieval on later memory. According to their account, during retrieval practice the reinstated context representation is strengthened and updated to include the context at the moment of recall. Since the updated context includes both the study context and recall context, it provides more cues to guide memory search on a future retrieval attempt (Siegel & Kahana, 2014). In contrast, when an item is restudied, the context representation is neither reinstated nor updated, thus there will be fewer and weaker retrieval cues available for a later retrieval attempt.

While the episodic context account offers a clear explanation for why and how retrieval benefits memory, the details of the account remain to be clarified. First, it is unclear *what* is reinstated during the memory retrieval. Context is a broad term and can refer to multiple features of an event (e.g., the modality of presentation, the speaker’s voice, the presentation font), as well as background features (e.g., the current location, the participants’ mood, background noises) (Bjork & Richardson-Klavehn, 1989; Geiselman & Bjork, 1980). Many memory models highlight temporal context as being especially important for memory retrieval. Temporal context is assumed to change slowly over time so that items that are encoded nearby in time share similar temporal contexts (Kahana, 1996; Polyn, Norman, & Kahana, 2009). Karpicke et al. (2014) emphasize the importance of temporal context, but no further details are given about what aspects of context are or are not encoded and reinstated during retrieval.

There is some evidence that temporal context is strengthened during retrieval practice. Participants who study multiple lists and are asked to retrieve the items after each list are better able to identify on which list an item occurred during a final test as compared to participants who complete some other distractor task after each list. That is, participants who practiced retrieving the items have better memory for the items’ temporal contexts (Brewer, Marsh, Meeks, Clark-Foos, & Hicks, 2010; Chan & McDermott, 2007).

However, it is still unclear if other types of context are strengthened by retrieval practice. To date, only two studies - Brewer et al. (2010) and Akan, Stanley, and Benjamin (2018) - have directly examined the effects of retrieval practice on non-temporal context memory. Both studies examined participants’ memory for incidental details from the original study phase after retrieval practice, but their results differ substantially.

First, Brewer et al. (2010) found that retrieval practice did not improve memory for context features of the items themselves. In their study, participants encoded two lists of words. Each word was presented visually and spoken by a male or female voice. After each list, participants either recalled the items or completed a distractor task. When asked to recall who presented each word on a final test, participants who had practiced retrieving the items were no better at identifying if the voice was male or female than participants in the control condition.

However, a recent study by Akan et al. (2018) did find evidence that retrieval practice improves memory for context features of the items themselves. Participants studied word pairs in one of eight locations circling the screen. During retrieval practice, participants were presented with one word of the pair in the center of the screen and were asked to recall the second word. On a final test, participants were more accurate at identifying the word pair’s original spatial location when they had previously retrieved the items as compared to when they restudied them. Thus, the current evidence surrounding the episodic context account is mixed. There is some evidence that retrieval practice does enhance memory for temporal context, but mixed results for contextual aspects of the item itself.

Across three experiments, we tested predictions from the episodic context account by examining participants’ memory for the initial study context after successful retrieval practice and comparing it to the memory of participants who restudied the same items. Specifically, we examined memory for source information (font color). During the initial study phase, cue-target pairs were presented in four different colors. Participants then either practiced retrieving the targets or restudied them and were later asked to remember the original font color they saw during the study.

If the episodic context account is true, and retrieval practice benefits memory by reinstating and updating the entirety of episodic context from the encoding phase, items that were successfully retrieved during practice should show enhanced memory for context details (i.e. font color) compared to those that were restudied. The successful retrieval practice should require participants to reinstate the prior episodic context and strengthen participants’ memory for that context. Therefore, we expect better color memory performance for items that were successfully practiced than those that were restudied.

## Experiment 1

### Method

#### Participants

Sixty-two adults (14 were male; mean age was 19.6 years) participated in exchange for course credit or US$10. Participation was limited to students who indicated on a pre-screening questionnaire that they were not color blind. One participant was excluded for not following the instructions during the study phase, leaving 61 participants in the analysis: 31 in the retrieval practice condition and 30 in the restudy condition. Participants were randomly assigned to one of the two conditions and were tested individually or in small groups of up to four people (each on their own computer).

#### Stimuli

Participants studied 40 word-pairs selected from a pool of Jacoby’s (1996) norms. Each item in the wordpool includes a cue, a fragment and two possible target words that complete the fragment (one typical and one atypical). For example, the cue-fragment pair “Flower - _a_sy”, could be completed with *daisy* (typical) or *pansy* (atypical). To ensure that participants were actively recalling from the original study episode during retrieval practice, only the atypical associates were selected as target words in our stimuli. As a result, guessing based on cues (e.g. Flower - _a_sy) would frequently lead to an incorrect response (e.g. daisy) as participants are likely to complete the fragment with the more typical associate.

Out of the 104 items in the norms, we selected 40 “cue - atypical target” word pairs that fit our two selection criteria; (1) the difference in completion base rate of the typical (e.g. daisy) and atypical (e.g. pansy) alternative was greater than 0.3 and (2) the atypical target had a completion base rate less than or equal to 0.2. This led to our atypical target words having a mean completion base rate of 0.09 compared to a base rate of 0.62 for the typical alternatives. By only using atypical items we aimed to ensure that correctly identifying a target word could only be achieved via episodic memory recall, not by guessing or generating from semantic memory.

#### Procedure

**Fig. 1:**
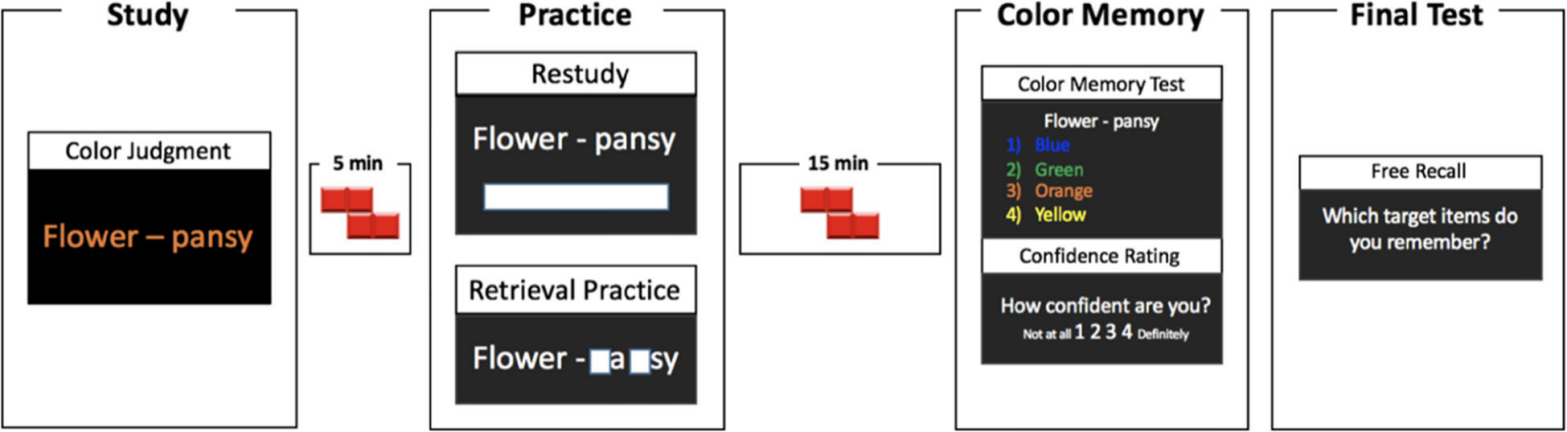
A schematic depiction of the design of Experiment 1. See text for details

A visual overview of the procedure is shown in Fig. 1. Participants were first given a brief overview of the experiment by doing a quick training round consisting of the study, practice, and final test phases with four word-pairs that were not used in the experiment. The color memory test was not included in the training round to make it less likely that participants would intentionally try to remember the font colors for the later test.

The main experiment began with the study phase, which included 40 cue-target pairs. Each word pair was presented in one of four colors (blue, green, orange, or yellow), with 10 cue-target pairs in each color. The order of the word pairs was randomized for each participant. Each pair was presented for 5 s with a 1-s interstimulus interval. During presentation, participants indicated the font color of each word pair by pressing a corresponding key on the keyboard (color stickers were placed over four of the keys).

A 5-min distraction period followed the study phase, during which participants played a video game (Tetris) on the computer. The key manipulation occurred during the next phase (practice). For participants in the restudy condition, the 40 cue-target pairs were presented in a scrambled order and they were asked to type the target word into a blank line under the target word. For the retrieval practice condition, participants were shown the cue word and the target fragment (again in a scrambled order). Participants were asked to complete the fragment by recalling the word they had seen earlier. For both the restudy and retrieval practice conditions, all words were presented in white font or on a black background.

After the practice phase, participants again played a video game (Tetris) on the computer for 15 min. Then, in the color memory test, participants were shown each of the pairs again and were asked to indicate in which of the four colors the pair was presented during the study phase. After each response, they indicated their confidence on a 4-point scale with 1 indicating “not confident at all” and 4 indicating “definitely confident”.

Finally, participants were asked to recall as many of the target words as possible, in any order (free recall phase). This final free-recall test occurred immediately after participants re-experienced all of the word pairs on the color test and thus is not a pure measure of the effects of retrieval practice on memory. Our primary goal was to assess participants’ memory of aspects of the study context. Thus, it was important to test color memory before free recall. Given that the color memory test involves another presentation of both the restudied and retrieval practiced items, it will tend to work against finding a canonical retrieval practice effect. In experiment 3, we reverse the order of the free recall and source memory tests to assess the robustness of our findings.

## Results

### Study phase

All participants successfully identified the font color of each word pair shown during study by pressing a key that matched in color (color indication accuracy *M* = 0.99, *SD* = 0.03).

### Practice phase

On average, fragment completion was successful on 51% of the trials (*SD* = 0.18).

### Color memory

According to the episodic context account, successfully recalling an item from a past study episode requires reinstating the entire context associated with the study event and (as a result) strengthening it. In our paradigm, the episodic context account predicts enhanced memory for font color when the items have previously been successfully retrieved. Thus, we compared color memory across the retrieval practice and restudy groups. In order to examine the effects of *successful* retrieval, we limited the analysis within the retrieval practice group to items that were successfully retrieved during the practice phase. While this analysis may suffer from item effects (the successfully retrieved items are likely the items that are easiest to remember), this should work in favor of the episodic context account - improving color memory for the items that were previously retrieved.

**Fig. 2:**
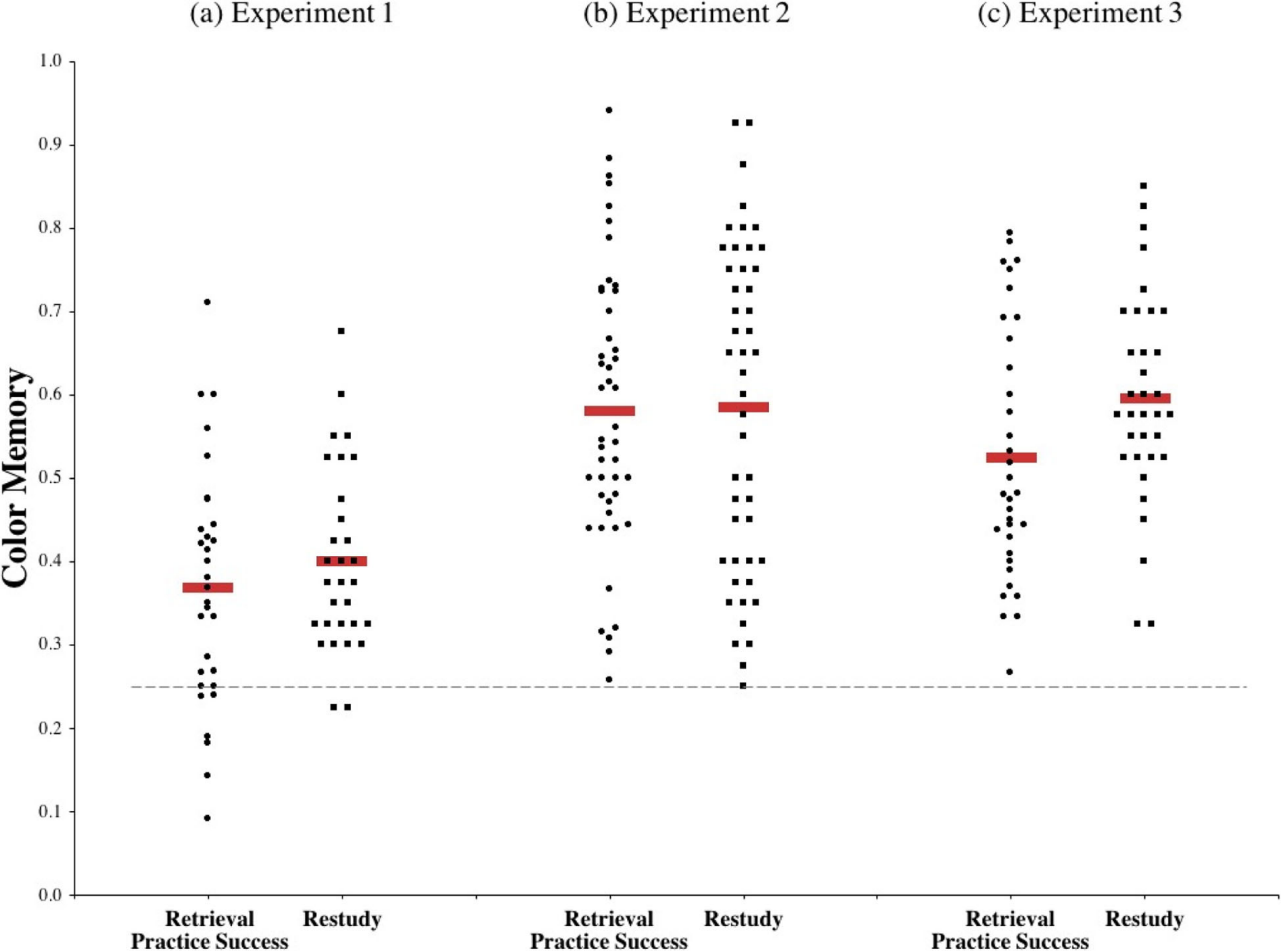
Proportion correct on the color memory test from (**a**) Experiment 1, (**b**) Experiment 2 & (**c**) Experiment 3. Across all three experiments, successful retrieval practice did not result in improved memory for source details from the original study event (font color). Each dot or square represents one participant. The red bars indicate the mean score in each condition and the dashed line indicates chance performance (0.25)

In contrast, we found that color memory for items that were successfully retrieved during practice (*M* = 0.37, *SD* = 0.14) did not differ from color memory in the restudy condition (*M* = 0.40, *SD* = 0.11), *t* (59) = 0.98, *p* = .33, *d* = 0.25, 95% CI [− 0.10, 0.03] (Fig. 2a). Color memory performance in both groups was well above chance levels (0.25). We conducted a Bayesian analysis to quantify the relative support for two hypotheses: one in which memory for font color is higher for successfully retrieved items as compared to restudied items (as predicted by the episodic context account), and a null hypothesis in which there is no difference between the groups (using JASP; JASP Team, 2018). Using a standard Cauchy distribution prior width of 0.707, we calculated an estimated Bayes factor (null/alternative) of BF_01_ = 6.87. In other words, the data were 6.87 times more likely to occur under the null hypothesis than the alternative hypothesis. A Bayes factor of 3 is typically considered moderate evidence and a factor of 10 is considered strong evidence (Lee & Wagenmakers, 2014).

#### Confidence ratings

Participants’ confidence ratings on the font color task were similar across participants who restudied the items (*M* = 2.21, *SD* = 0.48) and for the successfully retrieved items in the retrieval practice condition (*M* = 2.15, *SD* = 0.65), *t* (59) = 0.44, *p* = .66, *d* = 0.11, 95% CI [− 0.36, 0.23].^1^

### Free recall

When scoring the free recall data, simple typing errors were ignored (e.g. *thief* and *theif* were both counted as correct responses). Items that were successfully retrieved during the practice phase were later better remembered than items in the restudy condition (retrieval practice success, *M* = 0.35, *SD* = 0.15 versus (vs) restudy, *M* = 0.24, SD = 0.12), *t* (59) = 3.02, *p* = .004, *d* = 0.77, 95% CI [0.04, 0.18]. Items that were not successfully retrieved during the practice phase were less likely to be remembered during the free recall phase than items that were (retrieval practice failure, *M* = 0.19, *SD* = 0.14), *t* (30) = 5.94, *p* < .001, *d* = 1.07, 95% CI [0.11, 0.22].

## Discussion

Participants in experiment 1 showed no evidence of increased memory for context following retrieval practice. According to the episodic context account, broad contextual details from the initial study phase are reinstated during retrieval. Since participants were shown the words in different colors during the initial study phase, a globally reinstated context should include source details such as font color. However, participants did not have enhanced memory for the original study context following successful retrieval. Overall, the results from experiment 1 were inconsistent with the episodic context account of retrieval practice. We not only failed to observe enhanced memory for context for successfully retrieved items, but the overall trend was in the opposite direction with the restudy group showing better memory for context. Nevertheless, a more specific or targeted version of the episodic context account may still be true, in which a narrower slice of study context is reinstated to support retrieval of the target word.

In experiment 1, participants pressed a key to indicate the font color of the studied item. Encoding the font color was technically obligatory, but this encoding may have been shallow, and did not relate the font color to the identity of the study item. It is possible that details of the episodic context were indeed reinstated during retrieval practice, but that font color was poorly integrated into the study context, and as such was not preferentially reinstated. Thus, in experiment 2, we used a deeper encoding task that required integrating the target word and the font color. This was designed to force font color to be a more integral part of the study context. If the episodic context account is true, we should see improved memory for font color in the retrieval practice condition.

## Experiment 2

### Method

#### Participants

Ninety-four subjects (30 were male; mean age was 20.67 years) participated in exchange for course credit or US$10. None of the subjects had participated in experiment 1 and all indicated on a prescreening questionnaire that they were not color blind. One participant was excluded due to a software error, leaving 93 participants in the analysis: 46 in the retrieval practice condition and 47 in the restudy condition. The participants were tested individually or in small groups of up to four people (each on their own computer).

#### Procedure

The procedure was identical to experiment 1, except for the study phase. Instead of simply indicating the color of each word pair, participants were asked to judge how consistent the font color was with the target word. Consistency was rated on a 4-point scale with a response of 1 indicating “not consistent at all” and 4 indicating “highly consistent”. For example, “LAKE – pool” presented in blue was commonly rated as highly consistent.

## Results

### Consistency ratings and practice phase

On average, participants rated the words as being moderately consistent with their colors (*M* = 2.43, *SD* = 0.56). In addition, the responses were well-distributed along the rating scale. On average, each participant rated 26% of the word pairs as 1 s, 26% as 2 s, 26% as 3 s, and 22% as 4 s. During the practice phase, fragment completion was successful on 62% of the trials (*SD* = 0.15).

### Color memory

The use of the integrative encoding task caused source memory performance to increase overall relative to experiment 1 (Fig. 2b). However, there was again no difference in color memory between items that were successfully retrieved during practice (*M* = 0.58, *SD* = 0.17) and items from the restudy condition (*M* = 0.59, *SD* = 0.19), *t* (91) = 0.10, *p* = .92, *d* = 0.02, 95% CI [− 0.08, 0.07]. As in experiment 1, color memory performance was well above chance levels (0.25) for both conditions. A Bayesian analysis with the same parameters as in experiment 1 (H_1_: font color is higher for successfully retrieved items as compared to restudied items vs H_0_: no difference) indicated moderate support for the null hypothesis, BF_01_ = 4.93.

#### Confidence ratings

Consistent with their performance on the color memory test, participants’ confidence ratings for their font color responses did not differ between items that were previously successfully retrieved (*M* = 2.82, *SD* = 0.43) and those that were restudied (*M* = 2.81, *SD* = 0.52), *t* (91) = 0.10, *p* = .92, *d* = 0.02, 95% CI [− 0.19, 0.21].

### Free recall

Items that were successfully retrieved during the practice phase were later better remembered than items in the restudy condition (retrieval practice success, *M* = 0.43, *SD* = 0.19 vs restudy, *M* = 0.30, *SD* = 0.14), *t* (91) = 3.75, *p* < .001, *d* = 0.78, 95% CI [0.06, 0.20]. Items that were not successfully retrieved during the practice phase were less likely to be remembered during the free recall phase than items that were (retrieval practice failure, *M* = 0.24, *SD* = 0.23), *t* (45) = 6.11, *p* < .001, *d* = 0.90, 95% CI [0.13, 0.25].

## Discussion

We again found no evidence of enhanced memory for global episodic context following retrieval practice. Overall, compared to our results from experiment 1, the only difference between the findings of the two experiments lies in the strength of the memories. Participants from experiment 2 had enhanced performance in both the retrieval practice and restudy conditions relative to those from experiment 1 across all four measures (retrieval practice success, free recall, color memory, and confidence). This improvement in performance can be explained by the deeper processing of the study items with the modified encoding task. Despite the overall enhancement of memory for font color, retrieval practice success did not selectively enhance memory for this aspect of the study context. Memory for the original font color was equivalent across items that were successfully retrieved during retrieval practice and items in the restudy condition. This pattern of results is again inconsistent with the episodic context account.

In experiment 3, we switched the sequence of the two final tests, the color memory test and the free recall test, to better match the typical design of a retrieval practice experiment.

## Experiment 3

### Method

#### Participants

Sixty-eight subjects participated in exchange for course credit or US$10 (21 were male; mean age was 20.67 years) and were evenly split between the retrieval practice and restudy conditions (*N* = 34 in each). None of the subjects had participated in experiments 1 and 2 and all indicated on a prescreening questionnaire that they were not color blind. The participants were tested individually or in small groups of up to four people (each on their own computer).

#### Procedure

The procedure was identical to experiment 2, save the final phase where the task order was switched. Therefore, participants first recalled all of the target words they learned throughout the experiment (free recall) and then moved onto answering questions about the font color of each cue-target pair from the initial study phase (color memory test).

## Results

### Consistency ratings and practice phase

On average, participants rated the words as being moderately consistent with their colors (*M* = 2.45, *SD* = 0.50). In addition, the responses were well-distributed along the rating scale. On average, each participant rated 26% of the word pairs as 1 s, 26% as 2 s, 25% as 3 s, and 23% as 4 s. During the practice phase, fragment completion was successful on 56% of the trials (*SD* = 0.15).

### Color memory

Contrary to the episodic context account, participants who restudied the target items were better at recalling the original font colors (restudy, *M* = 0.60, *SD* = 0.13) as compared to the successfully retrieved items within the retrieval practice group (retrieval practice success, *M* = 0.53, *SD* = 0.15), *t* (66) = 2.10, *p* = .04, *d* = 0.51, 95% CI [0, 0.14] (Fig. 2c). A Bayesian analysis with the same parameters as in previous experiments found strong support for the null hypothesis, BF_01_ = 11.28.

#### Confidence ratings

Participants’ confidence ratings for their font color responses did not differ between successfully retrieved items (*M* = 2.64, *SD* = 0.50) and restudied items (*M* = 2.79, *SD* = 0.39), *t* (66) = 1.41, *p* = .164, *d* = 0.34, 95% CI [− 0.37, 0.06].

### Free recall

Items that were successfully retrieved during the practice phase were better remembered than items in the restudy condition (retrieval practice success, *M* = 0.29, *SD* = 0.13 vs restudy, *M* = 0.19, *SD* = 0.12), *t* (66) = 3.36, *p* = .001, *d* = 0.81, 95% CI [0.04, 0.16]. Items that were not successfully retrieved during the practice phase were less likely to be remembered during the free-recall phase than items that were (retrieval practice failure *M* = 0.05, *SD* = 0.08), *t* (33) = 11.62, *p* < .001, *d* = 1.99, 95% CI [0.20, 0.28].

## Discussion

Consistent with what we found in experiments 1 and 2, we again found no evidence of improvements in the memory for the study context (font color) following successful retrieval practice. In fact, participants who restudied the items had better color memory. It is clear that successful retrieval does not improve memory for all aspects of the encoded episodic context.

### General discussion

Our findings suggest some limitations and constraints on the episodic context account proposed by Karpicke et al. (2014). Their account suggests that the benefits of recall practice stem from reinstating the episodic context of the original study event. Successful retrieval strengthens the context memory associated with the target item, and the retrieved context is updated to include features of the recall context. Because this strengthened and updated context memory becomes an effective retrieval cue for later recall attempts, items that receive retrieval practice become more memorable than those that were simply restudied.

While this account provides a tenable explanation as to why retrieval practice benefits memory, the account does not specify the type of information reinstated from the episodic context. Context, in general, is a broad concept, and can refer to many aspects of the encoded event including characteristics of the target item itself such as typeface, stimuli color, and mode of presentation, as well as other features of the episode such as the testing room, outside noises, one’s mood or internal thoughts. In the current studies, we found no evidence that contextual details of the studied words themselves were strengthened following retrieval even though these contextual details were part of the encoding process and were remembered at above-chance levels during the color memory test. It remains possible, however, that a more limited version of the episodic context account may be correct; one in which *some but not all* aspects of the episodic context are reinstated during retrieval practice.

Our results suggest that contextual aspects of the item itself (e.g. its color) are not strengthened by retrieval practice. This is consistent with the results from Brewer et al. (2010) who found that retrieval practice did not affect participants’ memory of a different aspect of the item (the voice of the speaker). Building on the results of Brewer and colleagues, we found that these contextual details were not strengthened even when they were emphasized as a part of an encoding task. Thus, neither the original item color nor the presenting voice is retrieved and strengthened during retrieval practice.

However, as mentioned earlier, there is evidence that memory for certain kinds of context are strengthened during retrieval practice. Retrieval practice has been shown to improve memory for temporal context - specifying which list an item appeared on, and spatial context - specifying where an item appeared on the screen (Akan et al., 2018; Brewer et al., 2010; Chan & McDermott, 2007). Finally, aspects of the context that are explicitly retrieved are also strengthened. Participants in the aforementioned Brewer studies who were asked to both recall the word and the speaker’s gender during retrieval practice did have enhanced memory for the speaker’s gender on a later test (Brewer et al., 2010).

The episodic context account suggests that the benefit of retrieval practice is due to the reinstatement of study-period contextual details and the association of these details with practice-phase contextual details. However, it seems that certain contextual characteristics of the study event (voice gender and font color) are not more memorable following retrieval practice. These contextual details are clearly part of the memory for the study event, as they are remembered at levels well above chance when tested directly. In addition, retrieval practice failed to increase context memory both when the encoding of context was incidental (experiment 1; Brewer et al., 2010) and when contextual details were an active part of encoding task (experiments 2 and 3). One possible interpretation of these results is that the “context” defined in the episodic context account is more limited than previously suggested. It may be the case that retrieval practice only enhances memory for those aspects of the event that are specifically targeted by the retrieval probe or are automatically reinstated during retrieval. The current research suggests that memory for item-related characteristics of a study event (such as voice gender and font color) are less likely to benefit from retrieval practice than spatial and temporal characteristics.

However, it is also possible that the retrieval practice advantage is unrelated to the successful retrieval of episodic contextual details. It could be that episodic contextual information from the study phase is sometimes recalled during the practice phase, but this has no bearing on the enhanced memorability of items that are successfully retrieved during practice. By this account, certain episodic contextual details (such as spatial position or list context) may simply be details that are more likely to be retrieved despite not explicitly being cued, and other contextual details (such as voice gender or font color) are less likely to be retrieved spontaneously. In other words, while the observation of enhanced memory for source details is consistent with the episodic context account of retrieval practice, it is also consistent with alternative theories in which source details are sometimes retrieved, but are not mechanistically involved in the retrieval practice advantage.

Regardless of the true cognitive mechanism underlying the retrieval practice advantage, the current experiments provide an important demonstration that retrieval practice does not strengthen *all* aspects of episodic context.

## Supplementary information

**Additional file 1.** Online Supplement: Additional bayes factor analyses for Experiments 1, 2, & 3.

## Data Availability

The data supporting the conclusions of this article are available on git@github.com:vucml/ecarp.git. All materials are available from the corresponding author on request.

## Footnotes

1 For simplicity, we only report Bayes factors for our key analyses (color memory accuracy) in the main text. Bayes factors for all of the secondary analyses can be found in the Additional file 1.
