# Supplementary material for: Examining the episodic context account: does retrieval practice enhance memory for context?
Source: Cogn Res Princ Implic. 2019 Dec 18;4:46. doi: 10.1186/s41235-019-0202-3 (PMC6920270; doi:10.1186/s41235-019-0202-3)
Supplement: Supplementary file 1 — Additional file 1. Online Supplement: Additional bayes factor analyses for Experiments 1, 2, & 3. [file 41235_2019_202_MOESM1_ESM.docx]

**Online Supplement**

**Additional Bayes Factor Analyses for Experiments 1, 2, & 3**


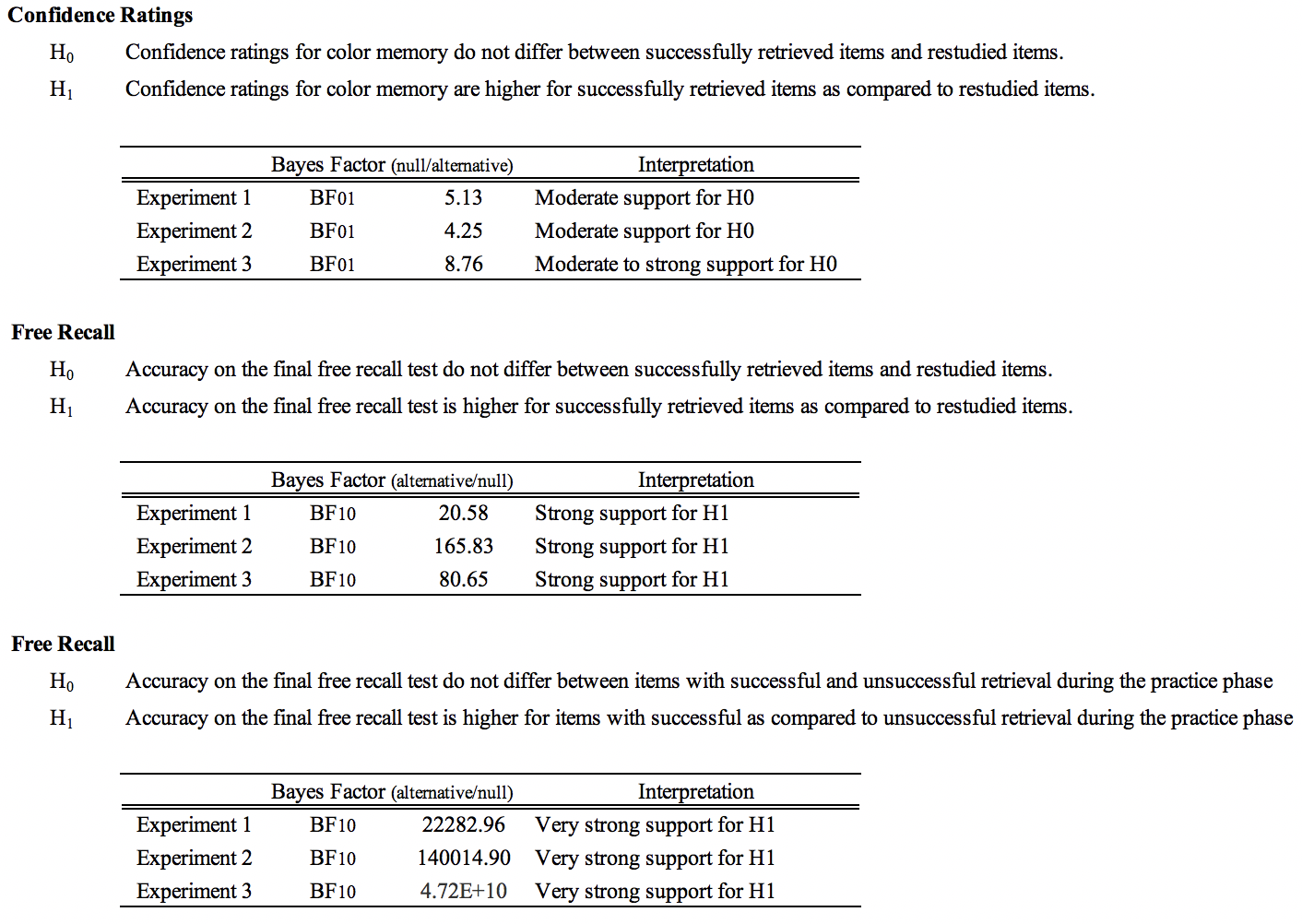
All of the Bayes Factor analyses reported here were conducted using JASP (JASP Team, 2018) with a standard Cauchy prior width of 0.707.
